# Intraocular Foreign Body: Diagnostic Protocols and Treatment Strategies in Ocular Trauma Patients

**DOI:** 10.3390/jcm10091861

**Published:** 2021-04-25

**Authors:** Hyun Chul Jung, Sang Yoon Lee, Chang Ki Yoon, Un Chul Park, Jang Won Heo, Eun Kyoung Lee

**Affiliations:** 1 Department of Ophthalmology, Seoul National University College of Medicine, Seoul National University Hospital, Seoul 03080, Korea; jhc042000@snu.ac.kr (H.C.J.); syst18@naver.com (C.K.Y.); ucpark@snu.ac.kr (U.C.P.); 2 Department of Ophthalmology, Seoul National University Hospital Biomedical Research Institute, Seoul 03080, Korea; forgotten100@gmail.com; 3 The One Seoul Eye Clinic, Seoul 06035, Korea; hjw68@snu.ac.kr

**Keywords:** intraocular foreign body, ocular trauma, open globe injury, prognostic factors

## Abstract

Intraocular foreign bodies (IOFBs) are critical ophthalmic emergencies that require urgent diagnosis and treatment to prevent blindness or globe loss. This study aimed to examine the various clinical presentations of IOFBs, determine the prognostic factors for final visual outcomes, establish diagnostic protocols, and update treatment strategies for patients with IOFBs. We retrospectively reviewed patients with IOFBs between 2005 and 2019. The mean age of the patients was 46.7 years, and the most common mechanism of injury was hammering (32.7%). The most common location of IOFBs was the retina and choroid (57.7%), and the IOFBs were mainly metal (76.9%). Multivariate regression analysis showed that poor final visual outcomes (<20/200) were associated with posterior segment IOFBs (odds ratio (OR) = 11.556, *p* = 0.033) and retinal detachment (OR = 4.781, *p* = 0.034). Diagnosing a retained IOFB is essential for establishing the management of patients with ocular trauma. To identify IOFBs, ocular imaging modalities, including computed tomography or ultrasonography, should be considered. Different strategies should be employed during the surgical removal of IOFBs depending on the material, location, and size of the IOFB.

## 1. Introduction

Intraocular foreign bodies (IOFBs) are defined as intraocularly retained, unintentional projectiles that require urgent diagnosis and treatment to prevent blindness or globe loss. IOFBs account for 16–41% of open globe injuries, and frequently cause severe visual loss in patients with ocular trauma [1,2,3,4]. Previous studies have described that the majority of IOFBs are work-related, and accidents are significantly more common among young men, who usually account for more than 90% of cases [5,6].

Ocular damage and visual loss may be caused by laceration or hemorrhage directly caused by IOFBs at the time of injury, but it may also occur due to the consequent development of retinal detachment or endophthalmitis. Various factors have been suggested to be associated with the final visual outcomes in patients with IOFBs. These factors include the initial visual acuity [7,8,9,10,11,12,13], size and location of the IOFB [9,11,12,13,14,15,16], size and location of the IOFB entry wound [7,12,14,17], presence of relative afferent pupillary defect (RAPD) [8,18], intraocular hemorrhage [8,12,18], retinal detachment [9,11,12,14,16,17,18], and endophthalmitis [10,12,15].

Confirming the presence or absence of a retained IOFB is essential for establishing the management of a patient with ocular trauma. In many cases, IOFBs may be identified by slit-lamp biomicroscopy or fundus examination. However, in some cases, it is difficult to identify IOFBs due to corneal damage or ocular media clouding, such as cataract, hyphema, or vitreous hemorrhage. Therefore, it is very important to carefully examine patients with IOFBs and utilize appropriate diagnostic techniques in specific situations. Most patients with IOFB require surgical treatment. The main objective of the treatment is to remove the IOFB, resolve complications that have already occurred, restore ocular anatomy, and minimize possible complications in the future. Depending on the patient’s condition, several staged operations may be required. Advances in microsurgical techniques have allowed for increased options in managing complicated cases [19,20,21]. Nevertheless, in some patients, preservation of the vision and globe may not be possible.

This study aimed to examine the various clinical presentations of IOFB and determine prognostic factors for final visual outcomes. Furthermore, we aimed to elucidate the diagnostic protocols and update the treatment strategy for patients with IOFBs presenting to an ocular trauma unit.

## 2. Materials and Methods

### 2.1. Participants and Clinical Assessment

This study was performed at the Seoul National University Hospital in Korea. The study adhered to the tenets of the Declaration of Helsinki and was approved by the Institutional Review Board of Seoul National University Hospital (IRB approval number: 2102-135-1199). We retrospectively reviewed the medical records of patients who were diagnosed with IOFB and underwent surgical removal of an IOFB between 2005 and 2019. The diagnosis of IOFB was confirmed by clinical examination, B-scan ultrasonography (USG), and/or computed tomography (CT).

The data collected included age, sex, mechanism of injury, initial best-corrected visual acuity (BCVA), initial ocular presentations, characteristics of the IOFB, time from injury to IOFB removal, interventions performed, complications, and final BCVA. The characteristics of the IOFB recorded included the location, material (metal, stone, glass, pencil lead, and other), and size of the foreign body. Endophthalmitis was diagnosed clinically based on the presence of hypopyon and vitreous opacities, anterior chamber inflammation, and increased pain.

All patients underwent comprehensive ophthalmic examinations, including measurement of BCVA, slit-lamp biomicroscopy, and indirect fundus examination. Fundus photography was performed using either a fundus camera (Vx-10; Kowa Optimed, Tokyo, Japan) or ultra-wide-field fundus photography (Optos 200Tx; Optos PLC, Scotland, UK) whenever possible. Patients most commonly underwent B-scan USG and a non-contrast CT scan of the orbits with thin slices if there was suspicion of a posterior segment IOFB. The BCVA measurements were converted to the logarithm of the minimal angle of resolution (logMAR) units before analysis. The following conversion to logMAR was used for vision worse than 20/2000: finger counting (FC) = 2.0, hand motion (HM) = 2.3, light perception (LP) = 2.6, and no light perception (NLP) = 2.9 [22].

### 2.2. Treatment Protocol

All patients with a leaking wound underwent primary repair of corneal or scleral lacerations performed by general ophthalmologists. If the IOFB was located in the anterior segment of the eye, removal was performed with primary globe repair. When the IOFB was located in the posterior segment of the eye, or associated with vitreoretinal complications, the patient was referred to the retinal unit for IOFB removal, and pars plana vitrectomy was performed. Lensectomy or phacoemulsification was performed in patients with traumatic cataract or lens injuries. A standard three-port vitrectomy (20-, 23-, and 25-gauge systems) was performed by a retinal specialist. Posterior vitreous detachment was carefully induced with an ocutome if one was not already present. IOFBs were extracted through an enlarged sclerotomy or limbal incision using intraocular forceps. External magnets or magnetic forceps were used in selected cases. Intraocular tamponade was performed using either expansile gas or silicone oil when the retina was damaged due to the IOFB or retinal detachment was present. In addition, endolaser photocoagulation was performed.

### 2.3. Statistical Analysis

Factors related to poor final visual outcomes were determined using univariate and multivariate linear regression analyses. Poor visual outcome was characterized by a BCVA worse than 20/200 at the last follow-up visit. Sex, characteristics of IOFB, clinical presentation, and management factors were nominal in scale and tested as independent factors using a regression model with dummy variables for male (1) and female (0), posterior (1) and anterior (0) IOFB locations, metallic (1) and non-metallic (0) IOFB, with (1) and without (0) vitreous hemorrhage, with (1) and without (0) retinal hemorrhage, with (1) and without (0) retinal detachment during the follow-up period, with (1) and without (0) endophthalmitis during the follow-up period, and with (1) and without (0) IOFB extraction with primary repair. Univariate and multivariate logistic regression analyses were used to identify the risk factors for poor visual outcomes. Variables with *p* < 0.15 in the univariate analysis were used for multivariate analysis in model 1. All variables were used for multivariate analysis with backward stepwise selection in model 2. All statistical analyses were performed using R software (version R, 4.0.4). Statistical significance was set at *p* < 0.05.

## 3. Results

### 3.1. Demographics and Mechanisms of Injury

The study included 52 eyes of 52 consecutive patients diagnosed with IOFB and managed with surgical removal of the IOFB. Table 1 summarizes the patient demographics and mechanisms of injury. The mean age of the subjects at the time of ocular trauma was 46.7 ± 15.8 years (range, 3–74 years). Fifty patients (96.2%) were men, and two (3.8%) were women. Overall, 22 patients (42.3%) had injuries in the right eye, and 30 (57.7%) had injuries in the left eye. The most common mechanisms of injury were hammering (17 eyes, 32.7%), followed by the use of an electric grass trimmer (13 eyes, 25.0%), drilling or grinding (seven eyes, 13.5%), cutting (three eyes, 5.8%), using a fishhook (two eyes, 3.8%), welding (two eyes, 3.8%), stabbing by a pencil (two eyes, 3.8%), car accidents (one eye, 1.9%), and miscellaneous (five eyes, 9.6%). At initial presentation, the visual acuity was 20/40 or better in 9 eyes (17.3%), 20/50 to 20/200 in 9 eyes (17.3%), 20/300 to FC in 13 eyes (25.0%), HM to LP in 20 eyes (38.5%), and NLP in 1 eye (1.9%). The most common initial BCVA was HM to LP (38.5%), followed by 20/300 to FC (25.0%). The mean initial logMAR BCVA was 1.60 ± 0.93.

### 3.2. Characteristics of IOFB and Clinical Presentation

**Figure 1:**
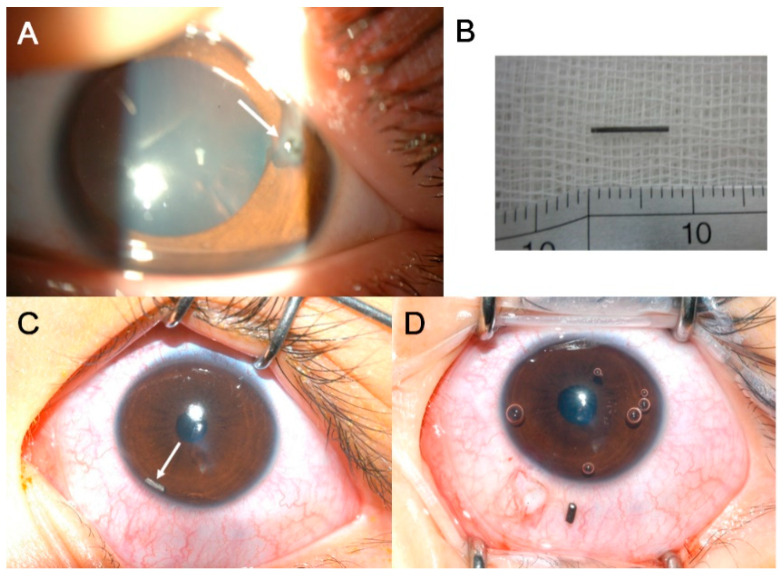
Anterior segment photograph of patients injured by mechanical pencil lead. (**A**,**B**) Left eye of an 8-year-old girl pricked with mechanical pencil lead while playing with a friend. The distal end of the lead is embedded in the iris and some of the lead is protruding from the cornea (*long white arrow*). The length of the removed mechanical pencil lead is 7 mm. (**C**,**D**) Left eye of a 9-year-old girl pricked with mechanical pencil lead. A piece of the lead is located in the anterior chamber (*long white arrow*), and the length of the removed mechanical pencil lead is 2 mm.

**Figure 2:**
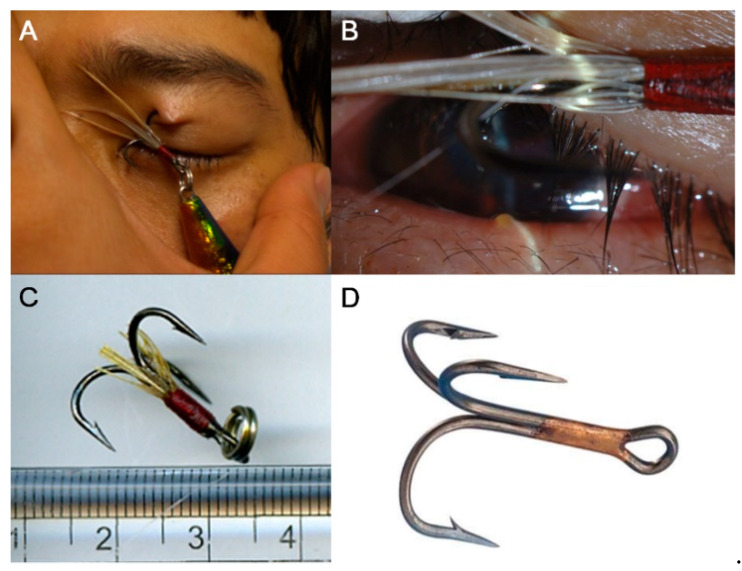
Anterior segment photograph of a 33-year-old male patient injured by a fishhook while pulling it during fishing. (**A**,**B**) One barb is embedded in the eyelid, and the other barb has pierced the cornea and is embedded in the iris and the anterior lens capsule. The removed fishhook (**C**) has a total length of approximately 2 cm, and the length of the barb is about 1 cm. It is a triple-barbed fishhook (**D**).

**Figure 3:**
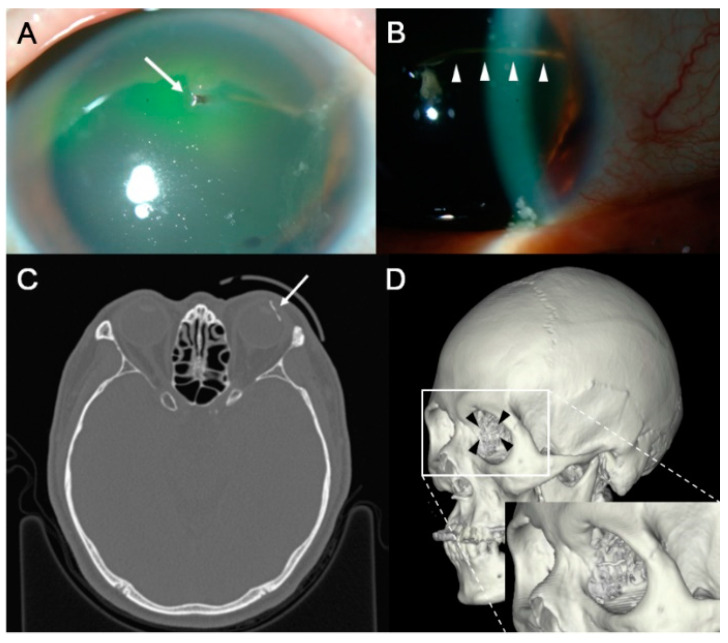
Anterior segment photograph and CT scan of the eye of a 60-year-old male patient who was pierced by a wire during work. (**A**,**B**) The distal end of the linear wire has reached the vitreous cavity through iridodialysis and zonule (*white arrowheads*), and the proximal end is exposed outside the cornea (*long white arrow*). On the axial CT scan (**C**), the direction of the wire (*long white arrow*) is successfully observed, and the 3D reconstructed image (**D**) shows the shape and position of the wire (*black arrowheads*), which is more remarkable on magnified images (*white box*).

**Figure 4:**
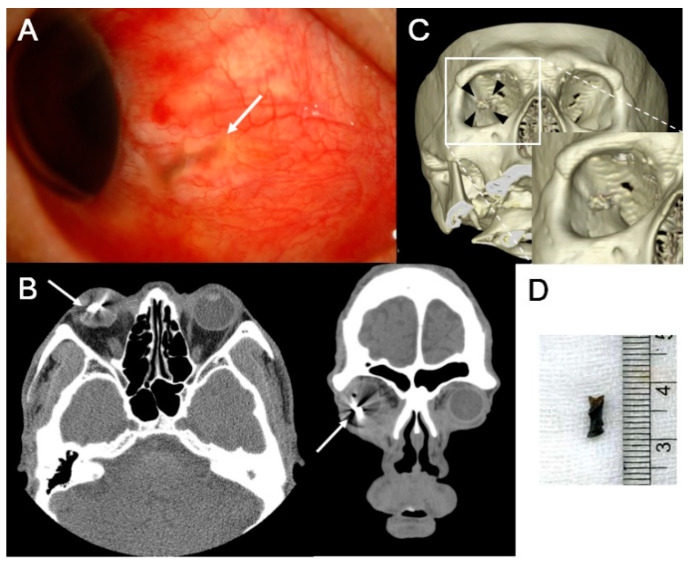
Anterior segment photograph and CT scan of a 54-year-old male patient struck by a metallic foreign body in his right eye during work. (**A**) A laceration site in the nasal sclera (*long white arrow*) is observed as an inlet of the intraocular foreign body (IOFB). (**B**) Axial and coronal CT scan of the patient shows the hyperdense foreign body (*long white arrows*) in the vitreous cavity and intraocular air. (**C**) On the 3D reconstructed image, the shape of the IOFB (*black arrowheads*) is better documented, which is more remarkable on magnified images (*white box*). (**D**) The length of the removed metallic foreign body is 7.5 mm.

**Figure 5:**
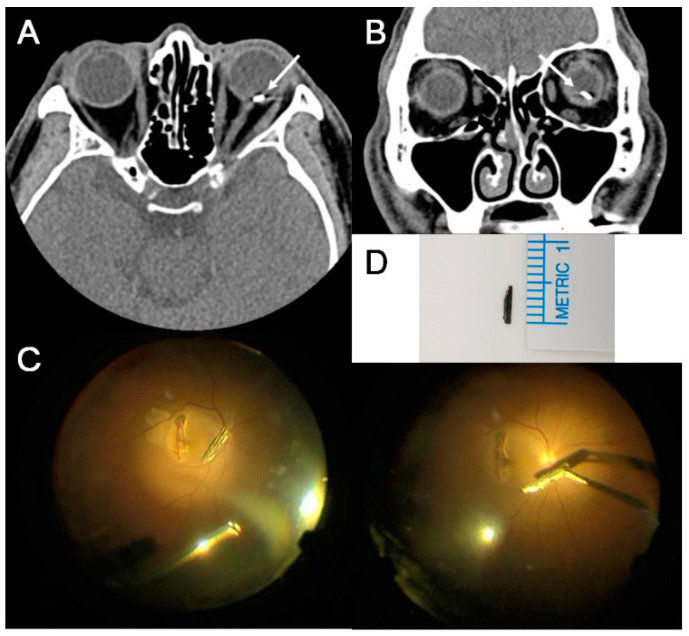
CT scan and intraoperative view of a 59-year-old male patient struck by a metallic foreign body in his left eye during work. (**A**,**B**) Axial and coronal CT images of the patient show a hyperdense foreign body (*long white arrows*) embedded in the retina. (**C**) Intraoperative view during vitrectomy shows a macular tear and an intraocular foreign body (IOFB). The IOFB was retrieved with vitreous forceps. (**D**) The length of the removed metallic foreign body is 5 mm.

**Figure 6:**
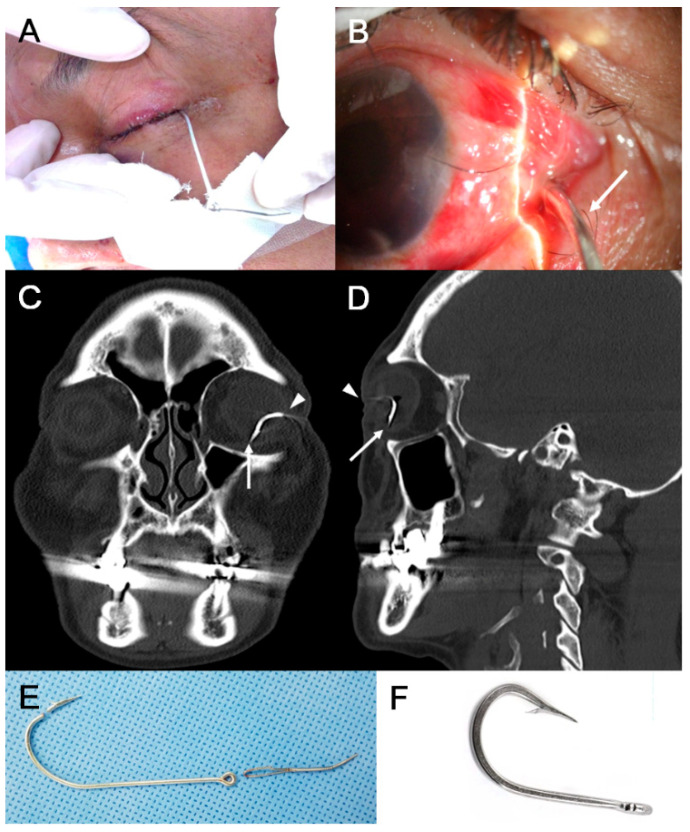
Anterior segment photograph and CT scan of a 54-year-old male patient injured by a fishhook while pulling it during fishing. (**A**,**B**) The fishhook (*long white arrows*) has penetrated the temporal sclera in the left eye, and the distal end of the needle is not identified. (**C**,**D**) Coronal and sagittal CT images of the patient showing the fishhook penetrating the eyeball (*white arrowheads*) and the double perforation into the attachment site of the inferior rectus muscle (*long white arrows*). (**E**) Since the fishhook cannot retrieved through the inlet due to the barb, it is moved forward, and the lower part of the barb is cut with a wire cutter. The remaining fishhook is removed by moving backward toward the inlet. (**F**) The removed fishhook is a simple single-barbed fishhook.

The characteristics of IOFB are summarized in Table 2. The IOFB was located in the cornea in four eyes (7.7%), embedded in the iris in six eyes (11.5%; Figure 1A,B), located in the anterior chamber in one eye (1.9%; Figure 1C,D), embedded in the lens in three eyes (5.8%; Figure 2), located in the vitreous in eight eyes (15.4%; Figure 3 and Figure 4), and embedded in the retina and choroid in 30 eyes (57.7%; Figure 5 and Figure 6). The most common location of IOFB was the retina and choroid in 30 eyes, followed by the vitreous in eight eyes. Accordingly, IOFBs were located in the anterior segment in 14 eyes (26.9%) and in the posterior segment in 38 eyes (73.1%). The site of penetration of the IOFB was the cornea in 31 eyes (59.6%), sclera in 9 eyes (17.3%), and corneosclera in 12 eyes (23.1%).

Of the 52 IOFBs removed, metallic IOFBs were noted in 40 eyes (76.9%), whereas non-metallic IOFBs including stone (five eyes, 9.6%), glass (three eyes, 5.8%), pencil lead (two eyes, 3.8%), and others (two eyes, 3.8%) were observed in 12 eyes (23.1%). The size of the IOFBs retrieved ranged from 0.5 mm to 30 mm (average, 5.16 ± 5.50 mm).

The initial clinical presentations are summarized in Table 3. The three most common clinical presentations were corneal injury in 43 eyes (82.7%), traumatic cataract or lens injury in 32 eyes (61.5%), and retinal tear in 32 eyes (61.5%). All included patients had phakia at the time of injury, and none had pseudophakia or aphakia. There were no patients with dislocation of the lens or intraocular lens (IOL), even though 32 patients presented with traumatic cataract or lens injury. Hyphema was noted in 12 eyes (23.1%), and iris injury was present in 25 eyes (48.1%). Of the 52 patients, we were unable to assess RAPD in 15 patients due to iris prolapse in corneal laceration or traumatic hyphema. Of the remaining 37 patients, 12 (32.4%) presented with RAPD. In addition, there was an extraocular muscle injury in one eye. Vitreous and retinal hemorrhages were noted in 25 eyes (48.1%). Retinal detachment was observed in 21 eyes (40.4%), and endophthalmitis was noted in four eyes (7.7%).

### 3.3. Management and Treatment Outcomes

The time to primary repair was 1.88 ± 4.44 days (range, 0–30 days). The IOFBs were removed during the first surgery in 45 eyes (86.5%). In seven eyes, IOFB removal was deferred due to a severely compromised view, failure to attempt to remove the IOFB, or lack of vitreoretinal expertise.

All patients underwent primary repair of the ruptured globe. The surgical management and treatment outcomes of eyes with IOFB are summarized in Table 4. The primary surgical procedures performed were anterior chamber washout in 6 eyes (11.5%), phacoemulsification of cataract in 9 eyes (17.3%), pars plana lensectomy in 18 eyes (34.6%), and pars plana vitrectomy in 33 eyes (63.5%). Of the nine eyes that underwent phacoemulsification of cataract during the primary surgery, posterior chamber IOL implantation was performed in two eyes, sulcus IOL implantation was performed in three eyes, and four eyes remained with aphakia, of which IOLs were implanted in the sulcus at a later stage in two eyes. All 18 eyes that underwent pars plana lensectomy during the primary surgery remained with aphakia, and IOLs were scleral fixated at a later stage in nine eyes. In addition to the repair of the ruptured globe with primary surgical procedures, the patients required additional surgeries during their follow-up. Additional surgical procedures performed were anterior chamber washout in two eyes (3.8%), phacoemulsification of cataract in four eyes (7.7%), pars plana lensectomy in three eyes (5.8%), pars plana vitrectomy in 18 eyes (34.6%), penetrating keratoplasty in three eyes (5.8%), scleral buckling in five eyes (9.6%), and glaucoma surgery in one eye (1.9%). Enucleation was not required (0%). The indications for the additional surgical procedures were as follows: anterior chamber washout for hyphema; phacoemulsification and lensectomy for traumatic cataract; vitrectomy for retinal detachment in eight eyes, vitreous hemorrhage in two eyes, silicone oil removal in three eyes, silicone oil removal with epiretinal membrane peeling in one eye, silicone oil removal with scleral fixation of IOL in one eye, scleral fixation of IOL in three eyes; penetrating keratoplasty for corneal opacity; scleral buckling for retinal detachment; and glaucoma surgery for Ahmed implant insertion for secondary glaucoma. The average number of surgical procedures performed per patient was 1.72 ± 0.83 (range, 1–4).

Systemic antibiotics were administered to all patients immediately at the time of diagnosis as part of prophylaxis for endophthalmitis. Intravitreal antibiotics were injected in selected patients based on the surgeon’s preference at the time of primary repair. Topical moxifloxacin was administered to all patients after the primary repair. Of the four cases of endophthalmitis among patients included in this study, all four occurred at the initial presentation, and none occurred during follow-up visits. Two patients received intravitreal antibiotic injections at the time of primary repair and were treated with systemic antibiotics. The other two patients were immediately treated with pars plana vitrectomy, intravitreal antibiotic injections, and systemic antibiotics. None of the patients underwent evisceration for infection control.

At the final visit, the BCVA was 20/40 or better in 17 eyes (32.7%), 20/50 to 20/200 in 10 eyes (19.2%), 20/300 to FC in 8 eyes (15.4%), HM to LP in 11 eyes (21.2%), and NLP in 6 eyes (11.5%). The final mean logMAR BCVA was 1.28 ± 1.13 after a mean follow-up period of 62.56 ± 71.29 months. Of the 52 eyes, four eyes (7.7%) progressed to phthisis bulbi and suffered globe loss.

### 3.4. Prognostic Factors

Univariate and multivariate regression analyses were performed using poor final visual outcomes (<20/200) as the dependent variable (Table 5). Among various clinical characteristics and management factors, initial visual acuity (odds ratio (OR), 2.913; 95% confidence interval (CI), 1.477–6.560; *p* = 0.004), posterior segment IOFB (OR, 25.000; 95% CI, 4.272–480.065; *p* = 0.003), vitreous hemorrhage (OR, 4.250; 95% CI, 1.374–14.230; *p* = 0.015), retinal hemorrhage (OR, 6.107; 95% CI, 1.915–21.606; *p* = 0.003), and retinal detachment (OR, 12.375; 95% CI, 3.470–54.025; *p* < 0.001) were associated with poor visual outcomes in the univariate logistic regression analysis. Multivariate logistic regression analysis showed that posterior segment IOFB (OR, 11.556; 95% CI, 1.695–234.465; *p* = 0.033) and retinal detachment (OR, 4.781; 95% CI, 1.186–22.428; *p* = 0.034) were significantly associated with poor visual outcomes.

## 4. Discussion

In developing countries, IOFBs are a serious problem in the young working-age population. In the present study, we analyzed the clinical presentation, characteristics, management and treatment outcomes, and prognostic factors related to poor visual outcomes of IOFBs at a single tertiary center. Consistent with previous reports [8,9,18], our study revealed that the majority of patients (96.2%) were male, with a mean age of 46.7 years. In the current study, 90.4% of the patients had work-related injuries, and hammering was the most common cause of injury (32.7%). Consistent with the findings of previous studies [13,23,24], metal was the most common material of IOFB, accounting for 76.9% of the patients, and IOFBs were most commonly located in the retina and choroid, accounting for 57.7%.

The energy transmitted to the eye by an IOFB is directly proportional to its mass and velocity [13]. Although the masses of IOFBs could not be measured in the current study, the mean size was 5.16 mm, which is quite large compared with the 3.5 mm size reported in a previous study [25]. Considering that as the IOFB size increases, its volume and mass also increase proportionally, the high proportion of posterior segment IOFBs in the current study (73.1%) may be attributed to the larger IOFB size.

**Figure 7:**
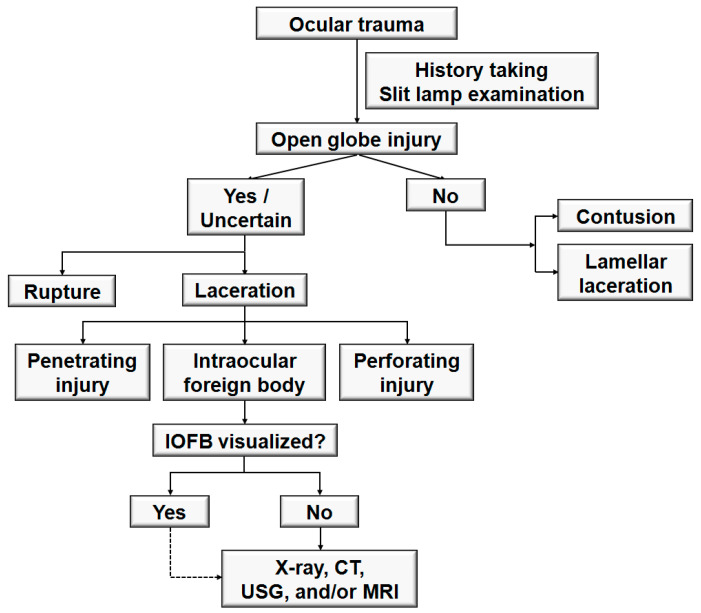
Flowchart for the diagnosis of intraocular foreign bodies. IOFB = intraocular foreign body; CT = computed tomography; USG = ultrasonography; MRI = magnetic resonance imaging.

Figure 7 shows the general thought processes that clinicians use when approaching patients with severe ocular trauma. A critical step in the diagnosis of IOFBs is the collection of a detailed history. The history should be thorough, and special attention should be paid to the mechanism of injury [1] and the setting in which the injury occurred. Knowing the mechanism of injury can enable clinicians to identify the nature and location of the IOFB and the accompanying ocular complications. Some patients do not experience pain or vision changes, even after ocular injury [26,27]. Children and bystanders are especially susceptible to being unaware of the injury and may present symptoms late [28]. Therefore, physicians should suspect an IOFB in all cases of open globe injury, including those who deny the possibility of IOFBs [25].

Kuhn et al. [29,30] recommended the Birmingham Eye Trauma Terminology (BETT) for clear definitions of all ocular injury types. Open globe injury is defined as a full-thickness wound in the corneoscleral wall of the eye. Closed globe injuries are subdivided into contusions and lamellar lacerations depending on the presence of a partial-thickness wound. Open globe injuries are subdivided into ruptures and lacerations. A rupture is a full-thickness wound of the eyewall, caused by a blunt object, whereas a laceration is a full-thickness wound of the eyewall, caused by a sharp object. In open globe injury with rupture, because the eye is filled with incompressible liquid, the impact results in a momentary increase in intraocular pressure. The eyewall yields at its weakest point at the impact site or elsewhere, such as an old cataract wound, and the actual wound is produced by an inside-out mechanism. In open globe injury with laceration, the wound occurs at the impact site by an outside-in mechanism [31]. From the point of view of physics, the kinetic energy (*E*) is determined by the mass (*m*) and velocity (*v*), using the equation *E* = 1/2 *mv*^2^. Blunt objects require higher kinetic energy to enter the eye (rupture) and are thus capable of inflicting more damage than sharp objects (laceration). Lacerations were further subdivided into penetrating injury (single entry wound), IOFB (retained foreign objects causing entrance laceration), and perforating injury (two full-thickness lacerations with entry and exit wounds).

After obtaining a detailed history, a complete ophthalmological examination, including an external inspection of the injury site, visual acuity measurement, slit-lamp examination, and fundus examination should be performed. Signs that suggest the presence or possibility of an open globe injury include the following [32]: obvious open wound (including positive Seidel test), collapsed or severely distorted eye, prolapsed uveal tissue, peaked pupil, subconjunctival hemorrhage with a shallow anterior chamber, and ocular hypotony with subconjunctival hemorrhagic chemosis.

Clinical examination alone cannot identify all IOFBs [4]. Therefore, ocular imaging modalities, including plain radiography, CT, magnetic resonance imaging, and USG, should be considered even when an IOFB is identified to rule out multiple IOFBs and aid in their accurate localization [13]. Plain radiography may be used for screening to detect and localize IOFBs or orbital foreign bodies [33]; however, as IOFBs that are not radio-opaque can be missed by this imaging modality, it is increasingly being replaced by CT [25]. The mainstay of radiologic evaluation is CT of the orbits without contrast [1,34]. It is the most reliable method for detecting IOFBs in patients with open globe injuries compared to clinical examination and B-scan USG [4]. Scanning in the sagittal and coronal planes with thin slices (1.0–1.5 mm) is required [35]. The limitations of CT include the possibility of missing certain ceramics, plastics, or wood [35]. Magnetic resonance imaging should be used only when the presence of a metallic IOFB is ruled out. This is because it can cause the movement of ferromagnetic IOFBs, leading to further intraocular damage [36,37]. B-scan USG can be used when an IOFB cannot be visualized directly or with a CT scan. However, it must be performed extremely carefully in selected eyes with open globe injury, because there is a risk of extruding the globe contents due to the pressure of the probe [38]. Although CT and B-scan USG are considered important diagnostic tools in establishing the diagnosis and treatment plan in patients with IOFB with ocular media clouding, anterior segment abnormalities could be evaluated using optical coherence tomography (OCT) with the corneal mode [39] or anterior OCT devices. Moreover, OCT is a critical diagnostic tool for assessing the posterior segment in patients with IOFB without ocular media clouding.

**Figure 8:**
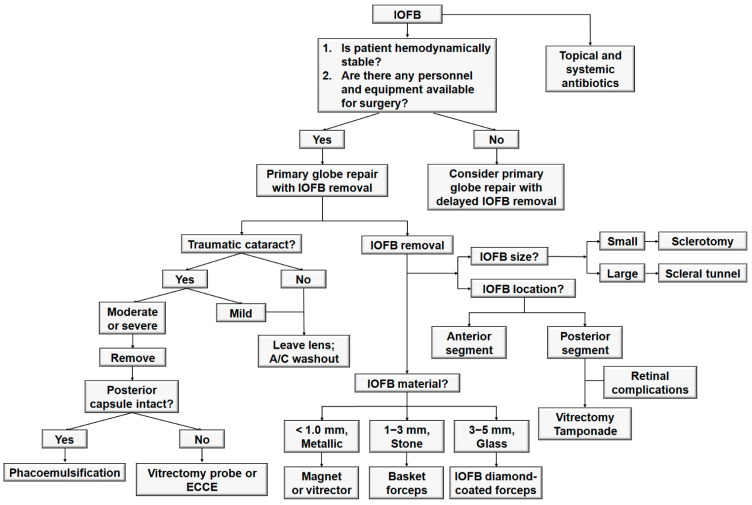
Algorithm for the management of intraocular foreign bodies. IOFB = intraocular foreign body; A/C = anterior chamber; ECCE = extracapsular cataract extraction.

Figure 8 illustrates strategic decision-making in managing eyes with IOFBs. Patients with IOFBs should be prescribed topical and systemic antibiotics immediately because the presence of an IOFB increases the risk of developing post-traumatic endophthalmitis [9,40,41]. Although the majority of cases of post-traumatic endophthalmitis are due to Gram-positive bacteria, empirical therapy with broad-spectrum intravenous antibiotics is the standard of care for the treatment of this condition [42]. The most common antibiotics used for post-traumatic endophthalmitis are vancomycin and ceftazidime [43]. Intravenous vancomycin is an excellent choice because it is effective against *Bacillus*, *Streptococcus*, and *Staphylococcus* species and has good intravitreal penetration [44,45]. Intravenous ceftazidime provides good Gram-negative coverage but is less effective than vancomycin in the coverage of *Bacillus* species [46]. Nevertheless, ceftazidime has a good safety profile and intravitreal penetration [47]. Intravenous ampicillin and sulbactam are alternative systemic antibiotics with good intraocular penetration and generally good Gram-positive coverage with variable coverage of *Bacillus* species [48]. In addition to systemic antibiotics, topical antibiotics are generally used, and intravitreal injection of antibiotics may also be considered during the initial repair in cases suspected to be at high risk for infection.

The timing of IOFB removal depends on several factors, including the patient’s general medical status, the composition of the IOFB, the nature of the injury, and the availability of operating equipment and trained personnel. If clinical signs of endophthalmitis are present, globe repair with immediate IOFB removal is almost always recommended, except when a simultaneous life-threatening injury precludes ophthalmic surgery [38]. If ophthalmologists who are experienced in the required surgery are not available, no definite signs of endophthalmitis are present, and the IOFB tends to be inert and well tolerated, or if the patient is hemodynamically unstable, it is advisable to delay IOFB removal and temporize with primary globe closure and administration of intravitreal and systemic antibiotics [1]. The patient should be referred to a specialist for definitive IOFB removal once the conditions become favorable.

Different strategies should be employed in the surgical removal of IOFBs depending on the material, location, and size of the IOFB. The extraction strategy for IOFBs is based on their size and material. Small (<1 mm), metallic, and ferromagnetic IOFBs may be removed with an intraocular magnet, whereas small, nonferrous materials can be removed with the vitreous cutter alone [1]. Intermediate-sized (1–3 mm) IOFBs may be removed with intraocular forceps or basket forceps, regardless of the material. Larger (3–5 mm) and glass IOFBs may require diamond-coated forceps designed to prevent slippage of the IOFB during removal [49]. Regarding the location of the IOFB, IOFBs from the anterior segment, including the cornea, anterior chamber, and intralenticular foreign bodies, are usually removed during primary globe repair, and intralenticular IOFBs are typically extracted together with cataract removal [25]. IOFBs in the posterior segment necessitate a careful analysis of the risks and advantages, and pars plana vitrectomy is usually required to remove posterior segment IOFBs. In cases where concomitant retinal complications, such as retinal tears and retinal detachment, occur or can develop as secondary consequences, an intraocular tamponade using gas or silicone oil is required. Regarding the IOFB extraction site, small and some medium-sized IOFBs can be removed through a sclerotomy with enlargement of the wound, if needed, whereas IOFBs larger than 4.0 × 4.0 × 4.0 mm^3^ may require scleral tunnel removal [1]. Following severe injury with large IOFBs, enucleation and evisceration are often considered as palliative treatment when all other therapeutic options are no longer effective, and patients either present with a severely injured eye that cannot be anatomically reconstructed or have a permanently blind and painful eye. With the development of orbital implants, the cosmetic outcome of enucleation approaches that of evisceration [50], although the latter may be more physiological.

The need for cataract surgery depends on the presence of intralenticular IOFBs or accompanying traumatic cataracts. If the IOFB is located in the cornea or anterior chamber and there is no or mild traumatic cataract, only the anterior chamber can be washed out and the lens may be left without surgery. However, if the IOFB is embedded in the lens, or if there is moderate or severe traumatic cataract, lens removal should be considered. The technique of lens removal depends on the area of no zonular support, integrity of the posterior capsule, and presence of vitreous prolapse. Favorable results are achieved with phacoemulsification if no vitreous prolapse is present and the posterior capsule and zonular structure are intact. However, lensectomy with a vitrectomy probe or extracapsular cataract extraction (ECCE) may be preferred if vitreous prolapse is confirmed or in case of extensive loss of zonular support or posterior capsular defects. Implantation of an IOL in an acute traumatic setting remains controversial. Although primary implantation has been performed successfully in selected cases [51,52,53], the risk of complications following a combined procedure should not be overlooked [54,55].

Multiple factors have been reported to be predictive of visual outcomes in patients with IOFBs. In our series, univariate analysis showed several predictive factors associated with poor visual outcomes, including poor initial BCVA, posterior segment IOFB, vitreous hemorrhage, retinal hemorrhage, and retinal detachment. Similar to previous reports [11,15], in the current study, the factors associated with poor visual outcomes in the multivariate analysis included a posterior segment IOFB. With anterior segment IOFBs, the injury tends to be limited to the anterior segment as the lens acts as a protective barrier, whereas with posterior segment IOFBs, serious complications such as retinal tears, retinal detachment, and proliferative vitreoretinopathy are common. However, even in the case of anterior segment IOFBs, if concomitant corneoscleral lacerations are present, behavioral abnormalities of the tear-film-free surface may lead to a loss of protection from ultraviolet light rays, and therefore predispose to various ocular diseases and contribute to poor visual outcome [56]. In our study, retinal detachment was also significantly associated with poor visual outcomes in the multivariate analysis. The removal of an IOFB in the presence of a detached retina is associated with an increased risk of iatrogenic retinal breaks, which in turn can lead to an increased risk of postoperative retinal detachment [48]. Several studies have reported that postoperative retinal detachment is associated with poor visual prognosis [11,12,14,16,17,18], although visual prognosis is even worse in the setting of preoperative retinal detachment.

This study had several limitations. First, this study was limited by its retrospective nature and the resulting non-standardized documentation, treatment, and follow-up. Second, our patient population was observed at a tertiary care referral center. Therefore, our results may not be generalizable to all patients with IOFB. Although we were able to minimize selection bias by including all consecutive patients seen during the observation period, we cannot exclude the possibility that referral and socioeconomic biases were present.

## 5. Conclusions

Ocular trauma continues to be a major cause of visual impairment. IOFBs constitute a significant component of ocular morbidity associated with open globe injuries. In this study, we evaluated the clinical presentation, characteristics, management, treatment outcomes, and prognostic factors of IOFB. Posterior segment IOFB and retinal detachment were significantly associated with poor final visual outcomes.

## Figures and Tables

**Table 1:** Demographics and mechanisms of injury of patients with intraocular foreign bodies.

Variable	Eyes with IOFB
Number of eyes	52
Age (yrs)	46.7 ± 15.8
Men/women	50/2
Right eye/left eye	22/30
Mechanisms of injury	
Hammering	17 (32.7%)
Electric grass trimmer	13 (25.0%)
Drilling/grinding	7 (13.5%)
Cutting	3 (5.8%)
Fishhook	2 (3.8%)
Welding	2 (3.8%)
Stabbed by the pencil	2 (3.8%)
Car accident	1 (1.9%)
Miscellaneous/unknown	5 (9.6%)
Initial VA	
≥20/40	9 (17.3%)
20/50–20/200	9 (17.3%)
20/300–FC	13 (25.0%)
HM–LP	20 (38.5%)
NLP	1 (1.9%)
Initial VA (logMAR)	1.60 ± 0.93

IOFB = intraocular foreign body; yrs = years; VA = visual acuity; FC = finger counting; HM = hand motion; NP = light perception; NLP = no light perception; logMAR = logarithm of minimal angle of resolution.

**Table 2:** Characteristics of intraocular foreign bodies (IOFB).

Variable	Eyes with IOFB
Location of IOFB	
Cornea, full thickness	4 (7.7%)
Iris	6 (11.5%)
Anterior chamber	1 (1.9%)
Lens	3 (5.8%)
Vitreous	8 (15.4%)
Retina/choroid	30 (57.7%)
Penetrating site	
Cornea	31 (59.6%)
Sclera	9 (17.3%)
Corneosclera	12 (23.1%)
Material of IOFB	
Metal	40 (76.9%)
Stone	5 (9.6%)
Glass	3 (5.8%)
Pencil lead	2 (3.8%)
Other	2 (3.8%)
Size of IOFB (mm)	5.16 ± 5.50

**Table 3:** Clinical presentations of eyes with intraocular foreign body (IOFB).

Variable	Eyes with IOFB
Corneal injury	43 (82.7%)
Hyphema	12 (23.1%)
Iris injury	25 (48.1%)
Traumatic cataract/lens injury	32 (61.5%)
Vitreous hemorrhage	25 (48.1%)
Retinal hemorrhage	25 (48.1%)
Retinal tear	32 (61.5%)
Retinal detachment	21 (40.4%)
Choroidal detachment	8 (15.4%)
Eyelid injury	4 (7.7%)
Endophthalmitis	4 (7.7%)

**Table 4:** Summary of surgical management and treatment outcomes of eyes with intraocular foreign bodies.

Variable	Eyes with IOFB
Trauma to primary repair (day)	1.88 ± 4.44
IOFB initially retrieved	45 (86.5%)
Primary surgical procedures performed	
Anterior chamber washout	6 (11.5%)
Phacoemulsification of cataract	9 (17.3%)
Pars plana lensectomy	18 (34.6%)
Pars plana vitrectomy	33 (63.5%)
Additional surgical procedures performed	
Anterior chamber washout	2 (3.8%)
Phacoemulsification of cataract	4 (7.7%)
Pars plana lensectomy	3 (5.8%)
Pars plana vitrectomy	18 (34.6%)
Penetrating keratoplasty	3 (5.8%)
Scleral buckling	5 (9.6%)
Glaucoma surgery	1 (1.9%)
Enucleation	0 (0%)
Number of surgical procedures performed	1.72 ± 0.83
Final VA	
≥20/40	17 (32.7%)
20/50–20/200	10 (19.2%)
20/300–FC	8 (15.4%)
HM–LP	11 (21.2%)
NLP	6 (11.5%)
Final VA (logMAR)	1.28 ± 1.13
Globe loss/survival	4/48
Follow-up period (months)	62.56 ± 71.29

IOFB = intraocular foreign body; VA = visual acuity; FC = finger counting; HM = hand motion; NP = light perception; NLP = no light perception; logMAR = logarithm of minimal angle of resolution.

**Table 5:** Univariate and multivariate logistic regression analysis for poor visual outcomes in eyes with intraocular foreign bodies.

Variables	Univariate Analysis		Multivariate Analysis			
			Model 1		Model 2	
	OR (95% CI)	*p*-Value	OR (95% CI)	*p*-Value	OR (95% CI)	*p*-Value
Age (yrs)	1.002 (0.967–1.038)	0.922				
Male sex (vs. female)	1.000 (0)	0.922				
Initial VA (logMAR)	2.913 (1.477–6.560)	**0.004**	2.096 (0.735–6.524)	0.174		
Characteristics of IOFB						
Posterior segment IOFB	25.000 (4.272–480.065)	**0.003**	6.066 (0.601–145.906)	0.164	11.556 (1.695–234.465)	**0.033**
Material of IOFB	4.059 (1.035–20.430)	0.058	0.448 (0.111–1.128)	0.190		
Size of IOFB (mm)	1.035 (0.935–1.163)	0.519				
Clinical presentations						
Vitreous hemorrhage	4.250 (1.374–14.230)	**0.015**	0.640 (0.058–5.509)	0.692		
Retinal hemorrhage	6.107 (1.915–21.606)	**0.003**	1.991 (0.206–20.573)	0.546		
Retinal detachment	12.375 (3.470–54.025)	**<0.001**	5.569 (0.966–44.478)	0.070	4.781 (1.186–22.428)	**0.034**
Endophthalmitis	1.000 (0.112–8.894)	1.000				
Management factors						
Trauma to IOFB removal time (day)	0.885 (0.605–1.044)	0.339				
IOFB extraction with primary repair	0.350 (0.047–1.812)	0.237				
Number of surgical procedures performed	1.704 (0.895–3.477)	0.118	0.550 (0.179–1.507)	0.261		

yrs = years; vs. = versus; VA = visual acuity; logMAR = logarithm of minimal angle of resolution; IOFB = intraocular foreign body; OR = odds ratio; CI = confidence interval. Bolded values represent significance, *p* < 0.05. Model 1 includes all of the risk factors with *p* < 0.15 in univariate analysis. Model 2 includes all of the risk factors selected by backward stepwise method.

## Data Availability

The data presented in this study are available on request to the corresponding author.
